# Droplet-Based Screening for the Investigation of Microbial Nonlinear Dose–Response Characteristics System, Background and Examples

**DOI:** 10.3390/mi11060577

**Published:** 2020-06-08

**Authors:** Jialan Cao, Felix Richter, Michael Kastl, Jonny Erdmann, Christian Burgold, David Dittrich, Steffen Schneider, J. Michael Köhler, G. Alexander Groß

**Affiliations:** 1 Institute for Chemistry and Biotechnologies, Department of Physical Chemistry and Microreaction Technologies, Technische Universität Ilmenau, 98693 Ilmenau, Germany; jialan.cao@tu-ilmenau.de (J.C.); felix.richter@tu-ilmenau.de (F.R.); steffen.schneider@tu-ilmenau.de (S.S.); michael.koehler@tu-ilmenau.de (J.M.K.); 2 CETONI GmbH Automatisierung und Microsysteme, 07554 Korbussen, Germany; michael.kastl@cetoni.de (M.K.); Jonny.Erdmann@cetoni.de (J.E.); Christian.Burgold@cetoni.de (C.B.); David.Dittrich@cetoni.de (D.D.)

**Keywords:** dose–response, droplet-based, segmented-flow, photo-fluorimetric, flow sensor, dynamic cultivation, screening, bacterial, microbial, proliferation, droplet generator

## Abstract

Droplet-based microfluidics is a versatile tool to reveal the dose–response relationship of different effectors on the microbial proliferation. Traditional readout parameter is the temporal development of the cell density for different effector concentrations. To determine nonlinear or unconventional dose–response relationships, data with high temporal resolution and dense concentration graduation are essential. If microorganisms with slow microbial growth kinetics are investigated, a sterile and evaporation-free long-term incubation technique is required. Here, we present a modular droplet-based screening system which was developed to solve these issues. Beside relevant technical aspects of the developed modules, the procedural workflow, and exemplary dose–response data for 1D and 2D dose–response screenings are presented.

## 1. Introduction

The investigation of the dose-response relationship is the objective of the most microbial screening programs. Microbiological culture screening plays an important role in different application fields. Microbiological cell assays are intensively used in context of medical [1], environmental [2,3], food [4], bioprocess or pharmaceutical [5] screening tasks. Important applications are e.g., the antibiotic susceptibility testing [6,7,8,9] of patient samples or profiling of new chemical compounds for antibiotic drug development [10,11,12]. The risk assessment of the growing number of antibiotic resistant organisms in the environment or hospitals requires dedicated sampling- and screening-strategies as well [13,14,15,16]. The investigation of the response behavior of complex microbial communities [17,18,19]—as they exist in soil or marine ecosystems—on toxic compounds or pollutants is a main topic in environmental research [20,21,22,23,24,25]. The eco-imperatively necessary changeover to a sustainable economy, forces the development of new bioprocesses that use bacteria as biocatalysts. The identification of new metabolic capacities from unknown bacteria and the investigation of the “biologic dark matter” is a hot research topic with high screening demands [17,26,27,28]. The isolation of rare organisms from the environment and the associated media optimization requires powerful screening methods to increase throughput and hit rates. A traditional dose–response experiment monitors the bacterial proliferation under varying effector concentrations. However, a good antibiotic substance inhibits the proliferation at low concentrations and a low IC50-Value will be found. Low active compounds require a high dose to suppress growth or the growth cannot be stopped at all. If end–point assay protocols are used, the cell density is determined after a fixed incubation time and the correlation between cell density and effector concentration reveals the minimum inhibitory concentration (MIC, IC50). This strategy works well if organisms with known proliferation kinetics are screened against different effector or media variations. In many of the more sophisticated screening approaches mentioned above, the organisms are unknown or consortia of organism are in the focus. For many of these examples, nonlinear or unconventional temporal dose–response relationships can be expected. Here, fixed end-point assays will lead to statistically insignificant results. Microfluidic droplet-based screening technology can help to solve this problem.

Recent advances in droplet-based screening proved this technique as powerful tool for appropriate microbial screening tasks [18,29,30,31,32,33,34]. Most important readout is the proliferation rate determined by optical measurements. The droplet-based screening approaches make use of small volume droplets which are confined by the device- or tubing-walls on one hand, and an immiscible carrier medium on the other. In this way, individual samples can be compartmentalized and handled in a serial manner as droplet sequences (segmented flow). The advantageous properties of droplet-based systems are: low individual sample volumes, fast mixing and fast processes for sample generation and processing, sterile conditions due to the closed environment, good control of the oxygen saturation depending on the oxygen permeability of the used materials, dense data depending on the adjusted composition change between the subsequent droplets. Droplet-based screening systems are well suited for long-term incubation, because of the sterile, closed environment and evaporation control. However, droplet-based systems are restricted to homogenous or dispersed cultivation conditions.

## 2. Dose–Response Screening Background

**Figure 1:**
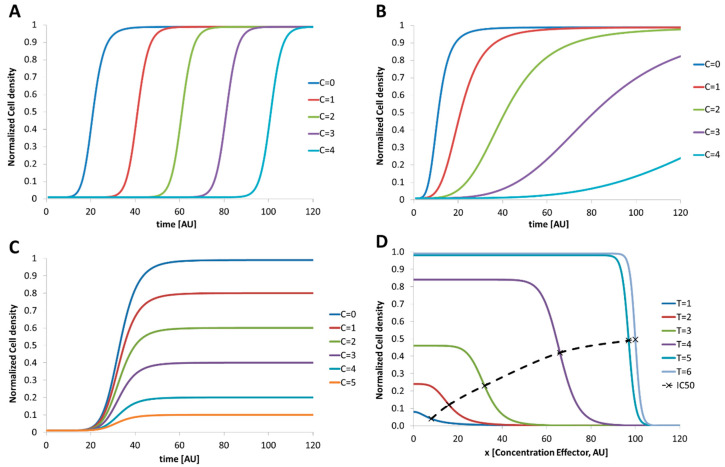
Schematic graphs indicate the development of the cell density over time in dependence on increasing effector concentrations. Examples for the different response types are: (**A**) increasing effector concentration prolongs the lag phase, but do not interfere with the other phases. Here, the organism needs some time to adapt to the changed conditions before the number of organisms starts to grow. Hence, the lag phase is proportional to the effector concentration; (**B**) increasing effector concentration reduces the exponential growth (doubling time). The organism number increase, but the effector reduces the rate of proliferation; (**C**) increasing effector concentration does not influence the lag—or exponential—phase but limits the maximum cell density in the stationary phase. Here, the number of organisms increases up to a limit proportional to the effector concentration. Depending on the chosen detection sensor for the cell density a change in morphology (organism may form aggregates or shrink as response to the effector) can cause this effect; (**D**) dose–response plot indicates the development of the cell density over the effector concentration for different times. It represents the data which are gained by experiments to derive the growth kinetics as shown before. Characteristic point is the inhibitory concentration which indicates the effector concentration that inhibits the growth by 50% of the maximum cell density without effector (IC50-Value). Dashed line indicates the temporal development of the IC50 values which would be determined at the different points in time.

**Figure 2:**
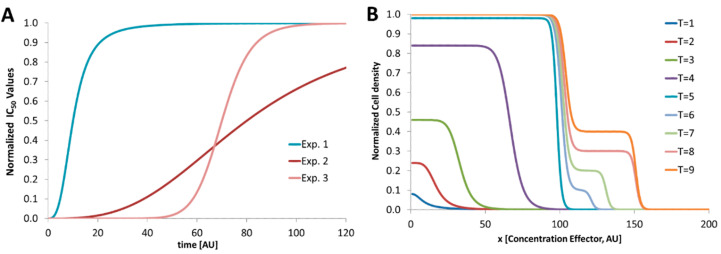
Schematic graphs. (**A**) Schematic development of IC50 values with respect to the varying response behavior to different experimental conditions such as different organisms, effectors or broth media; (**B**) nonlinear behavior of an organism which responds to the effector differently. After prolonged incubation (T > 5) a second growth phase takes place at higher effector concentrations (>100) and moves the IC50 value from about 100 to about 150 (AU).

**Figure 3:**
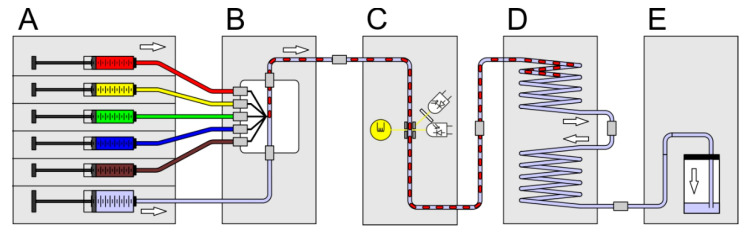
Schematic setup assembled for the generation of droplet sequences using a combination of different modules (**A**–**E**). Individual modules are connected by transparent FEP-tubes and standard fluidic connectors. (**A**) Syringe pump with six channels for fluid actuation (www.cetoni.de); (**B**) droplet generator; (**C**) photo-fluorimetric sensor module to record the initial photo-fluorimetric reference signals; (**D**) droplet sequence storage and incubation coil; (**E**) void volume.

In the following passage we will discuss a variety of possible dose–response relationships from a theoretical point of view, if non-linear or unconventional temporal dose–response relationships are expected. For microbial assays, dose–response experiments usually monitor the growth of a starting cell number up to dense cultures in dependence on different effector concentrations. In general, cell culture growth passes through four subsequent phases [35]: (1) Lag phase. No increase in the cell number can be seen in this phase. After inoculation, the organism has to adapt their metabolic functions to the new conditions (2) Log or exponential phase. If the adaption was accomplished, the number of cells increases exponentially. The time which is required to double the number of cells is defined as the doubling time or generation time. (3) Stationary phase. The cell density reaches a maximum and no more growth can be observed. In this state, the further proliferation is limited by the availability of nutrition or excreted metabolic products. The organism changes its metabolism to a survival mode, and some develop persistence forms. ((4) Death phase. Here, the number of living cells starts to decrease because of lack of nutrition or excreted toxic metabolites which kills the cells. The required dose of an effector which is necessary to omit microbial proliferation is determined by a dose–response assay. Therefore, different batches of the investigated microorganism are treated with increasing amount of the effector and the temporal development of the cell number is monitored. If microorganisms are investigated with different doses of effectors (C=0 < C=1 < C=2 < C=3) the proliferation can be influenced in three different ways: (1) The lag phase is prolonged, but the organism proliferates thereafter without restrictions (Figure 1A); (2) The organism proliferates but in dependence on the effector concentration with different growth rates in the exponential phase (Figure 1B); (3) The organism proliferates but the maximum cell density in the stationary phase depends on the effector. A similar behavior will be observed if the cell morphology is changed by the effector. In this case, optical detection can lead to a lower signal because of the changed scattering cross section of the cells (variation in size or aggregation; Figure 1C). However, a combination of all these effects or even stimulated growth or abnormal behavior may be induced by different effectors. Typical result of a dose–response experiment for a regular behavior is the 50% inhibitory concentration IC50. The IC50 value is defined as the reversal point in the growth curve and depends on the measurement time point as shown in Figure 1D. Here, the cell densities in dependence on different effector concentrations are shown for different time points. The associated IC50 value increases in time and reaches a limit (dashed line). The accurate determination of the IC50-value is mandatory if the investigated effector should be used to suppress the microbial proliferation. However, if unknown organisms or unconventional cultivation conditions are used the temporal IC50 development as shown in Figure 1D has to be recorded carefully. Static end point assays using a fixed incubation time may lead to wrong results because the organism still grows with low proliferation rates.

In Figure 2 the development of IC50 values for varying experimental conditions is shown. In Exp. 1 the IC50 values reach a steady state earlier than Exp. 2. Here, a steady IC50 is reached later because of a prolonged lag phase. Exp. 3 shows the development of the IC50 values when the generation time is prolonged by the effector. For Exp. 2 either the observation time is too short or the effector does not suppress the growth at all. However, for screening programs with such varying microbial growth conditions, droplet-based microfluidics can help to screen the time-dependent IC50 values in a fast and efficient way. For a more complex response of nonlinear growth behavior the response cannot be described by a plain parameter such as IC50. Here, the dose–response curve has to be considered. However, because of the varying response behavior the determination of inhibitory concentrations (IC50) depends strongly on the used microorganism, effector and experimental conditions. Especially the chosen medium has a strong influence and has to be assayed for each variation. If the investigated microorganism shows a complex behavior, the kinetic analysis is mandatory. In Figure 2B a nonlinear response is depicted. Here, the microorganism grows first like a usual kinetic (T = 1 to T = 5). But after a prolonged incubation (T = 6 to T = 9) a second growth phase at higher effector concentrations takes place. The microorganism proliferates with a prolonged lag phase and different morphology. However, for an effective growth inhibition an effector concentration higher than 150 (AU) is required.

Disadvantage of the determination of the optical density as screening readout is that there is no information about the fraction of dead cells in the sample. Dead cells may contribute in the same way to the signal as living cells. Even the change in the organism morphology under stress leads to a change in the absorbance signal and does not fit to calibration curves which are generated by dilution of a dense culture. Especially bacteria tend to form biofilms or aggregates when exposed to stress. However, if the proliferation of cultures is studied in dependence on different effector concentrations this boundary condition has to be considered. However, in most dose–response experiments the growth inhibition gives a valuable response for the desired assay task.

## 3. System Setup and New Developed Modules

For droplet-based dose–response screening campaigns a modular system was used. In general, a droplet-based screening process requires three operational steps: (1) generation of a droplet sequence with precise composition; (2) incubation and repeated online measurement of the cell density; (3) signal processing and data analysis. The modular system has the advantage of flexible assembly related to the desired fluidic protocols and readout wavelengths. The concept of modular microfluidic assemblies has been proven to be utterly practical in numerous publications before [36,37,38,39].

In Figure 3 the setup scheme for the generation of initial screening sequences is shown. The system comprises of the following modules: six channel syringe pump (A), droplet generator (B), micro flow-through photo-fluorimetric sensor unit (E), sequence storage module (D) and a void volume flask (C). For the generation of droplet sequences composed of droplets with increasing effector concentration, but equal bacteria/cell number and constant media composition the syringes must be loaded with the different solutions and actuated by appropriate controlling software. This way, sequences with one (1D) or two (2D) varying effectors can be generated. The received sequence will be in the upper part of the storage module and can be disconnected from the flow path by releasing the standard fluid connectors. If the storage coil ends are interconnected, the coil can be moved to another system or incubated in an incubator. The requirements for the syringe pump are very high, because the precision of the syringe pump directly influences the accuracy of the droplet composition, flow rates and time delays. Even the volume of the used syringes and the number of required channels must be regarded.

**Figure 4:**
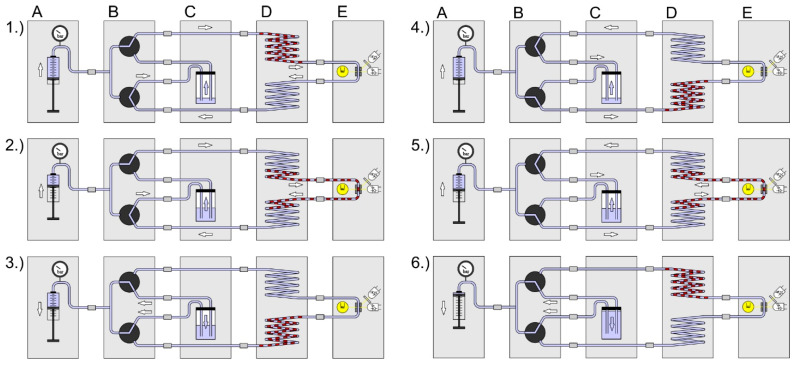
Setup scheme and operational sequence for the iterative measurement of droplet sequences. Modules: A: syringe pump; B: valve module; C: void volume; D: droplet sequence storage module; E: photo-fluorimetric sensor. Operational sequence: (**1**) After setup initiation, the droplet sequence is in the upper part of the storage module; (**2**) The (A) syringe pump dispenses fluid and pushes the droplet sequence through the (E) sensor; The (C) void volume container is filled by the carrier medium; (**3**) The droplet sequence is in the lower part of the (D) storage module and the (B) valve block is switched to aspirate carrier medium from the (C) void volume back into the (A) syringe; (**4**) When the (A) syringe is refilled the (B) valve module is switched to this flow path; (**5**) The (A) syringe delivers fluid and pushes the droplet sequence from the lower part of the (D) storage module through the (E) sensor into the upper part of the (D) storage module; (**6**) The droplet sequence is in the upper part of the (D) storage module and the (B) valve block is switched to aspirate carrier medium from the (**C**) void volume back into the syringe; When the syringe is refilled the sequence restarts at (**1**).

To reveal the kinetic dose–response relationships, the droplet sequences must be incubated and analyzed iteratively, and the cell density must be recorded for each individual droplet. Therefore, the sequence has to be pumped through the sensor module in defined time intervals. In Figure 4 the assembled setup scheme is shown. The following modules were combined: (A) syringe pump system, (B) valve module, (C) carrier container, (D) sequence storage module and (E) the photo-fluorimetric sensor module. The figure sequences 1–7 illustrate the screening process: (1) Initially the syringe pump (A) is filled, the container (C) is empty and the droplet sequence is in the upper part of the storage module (D). (2) The syringe pump delivers carrier medium and the valve module directs the flow path along the way through the storage coil upper part, the sensor module and the storage coil lower part into the carrier container. (3) When the droplet sequence has passed through the sensor completely, the syringe is empty, the container is filled, and the droplet sequence is placed in the lower storage coil. (4) The syringe aspirates the carrier medium from the container by switching the valve module to the appropriate flow path. (5) After the desired incubation time is over, the valve module changes the flow path in the opposite direction. (6) The syringe delivers carrier medium and the droplets are moved from the lower part of the storage coil through the sensor into the upper storage part. (7) The syringe pump is refilled with carrier medium from the container by appropriate adjustments of the valve module. This way, the growth kinetics of each droplet can be determined in an automated way. The generation of droplet sequences requires—in dependence on the complexity of the assay protocol—five to six individual syringe pump channels. However, only one syringe pump and the valve module are required for the kinetic measurement.

**Figure 5:**
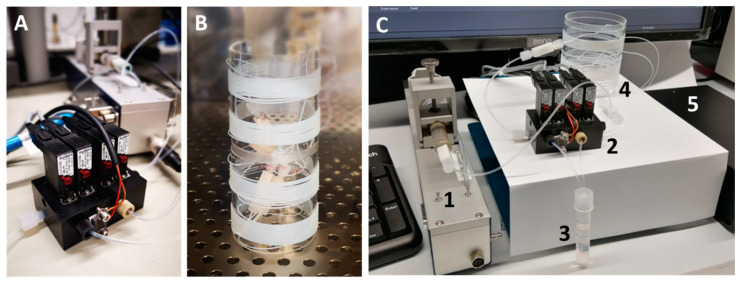
(**A**) Picture of the valve module required for dynamic cultivation using a single syringe pump; (**B**) storage module for droplet sequences located inside an incubator; (**C**) complete setup, including (1) syringe pump, (2) valve module, (3) void volume container, (4) storage coil and (5) photo-fluorometric sensor.

In Figure 5A the valve module is shown. The valve module consisted of four valves (model description: Bürkert 6144, Christian Bürkert GmbH & Co. KG, Ingelfingen, Germany) and a pressure sensor (model description: Merit Sensor TR1-0100G-101, Merit Medical Systems, Inc., South Jordan, UT, USA) for process control. The maximum operating pressure of the sensor and the valves was approx. 7 bar. Materials in contact with the medium are made of polyoxymethylene (POM), polyphenylene sulfide (PPS) and ethylene–propylene–diene rubber (EPDM). The maximum flow rate is limited by the back pressure of the entire system. Hence, the minimum time span for the analysis of a sequence is determined by the chosen capillary length and diameter. E.g. for a 5 m tubing (ID 0.5 mm) carrying about 1000 droplets the analysis time at 200 µL/min is about 5 min. However, for long term incubation experiments (up to 14 days) the analysis time is no limiting factor. For the time-dependent screening experiment, a flow rate of 30 µL/min was chosen, whereby a 10 m (2 × 5 m) long tubing with inner diameter of 0.5 mm has an average pressure of 0.5 bar. Incubation and storage were done in the storage coils as shown in Figure 5B.

The inoculum cell density is a crucial parameter for proliferation assays and must be chosen carefully. For droplet-based dose–response screening, a compromise between droplet volume and sensor sensitivity must be chosen. Low droplet volumes in nL or pL range are advantageous if high concentrations of excreted metabolic compounds are of interest. However, if only a low number of cells or even single cells are desired the Poisson distribution must be considered [40,41,42]. However, for antimicrobial susceptibility testing, e.g., initial densities of about 10^6^ cells/mL of the appropriate bacteria are recommended. 10^6^ cells/mL matches with 500 cells per 500-nL droplet. Here the Poisson distribution can be neglected. In Table 1 the cell density and the corresponding cell numbers in different volumes are given. Depending on the microorganism and used medium, cultures can proliferate unrestrictedly up to 10^9^ to 10^10^ cells/mL. This equals a final cell number of about 500.000 to 5 × 10^6^ cells per 500-nL droplet. In this scenario, the investigated microorganism can double its number up to 16 times until the stationary phase is reached. On the other hand, the photo-fluorimetric detector capabilities determine the minimal detectable cell numbers. Hence, we developed a system for a screening volume of about 500 nL.

If small droplets in the sub nL range are chosen, the number of cell division steps is limited by the available volume and media. Excreted metabolites can inhibit the growth or cause quorum sensing effects because the concentration increases fast in the low volume. We usually choose about 500 up to 5000 cells per 500-nL droplet. This equals a cell density of about 10^6^–10^7^ mL^−1^, which usually ensures enough media available to prevent self-inhibition in the early division cycles.

**Figure 6:**
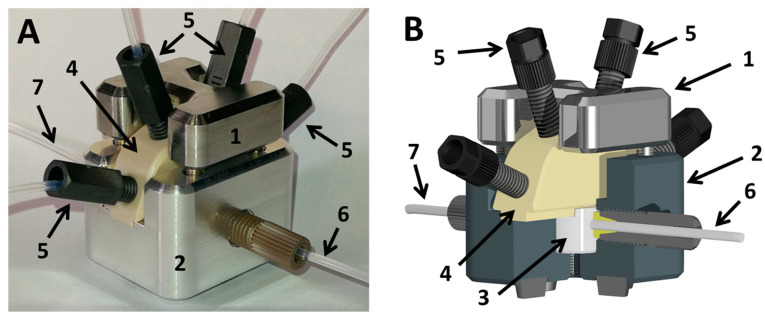
(**A**) Droplet generator (Figure 3B); (**B**) scheme of the droplet generator with sectional cut. (1) supporting frame top; (2) supporting frame bottom; (3) exchangeable PTFE inlet for droplet generation; (4) fluid manifold routing up to four different media streams into the PTFE droplet generator; (5) fluid connector for media supply; (6) fluid port for the carrier medium inlet; (7) fluid port outlet for the droplet sequence.

In Figure 6 the droplet generator is shown. The device was made by precision machining and designed with an exchangeable PTFE-inlay. The PTFE-inlet with different bore diameters allows the generation of a range of defined droplet sizes between 350 nL up to 1,200 nL. The device can be disassembled for ultrasonic cleaning and autoclaving. The precision of the screening data depends on various parameters. However, main factor is the accuracy of the droplet formation and the received precision of the droplet composition according to the desired flow protocol. To prove the capabilities of the overall system, the setup including the droplet generator was analyzed beforehand with dye solutions for validation for each fluidic actuation program.

**Figure 7:**
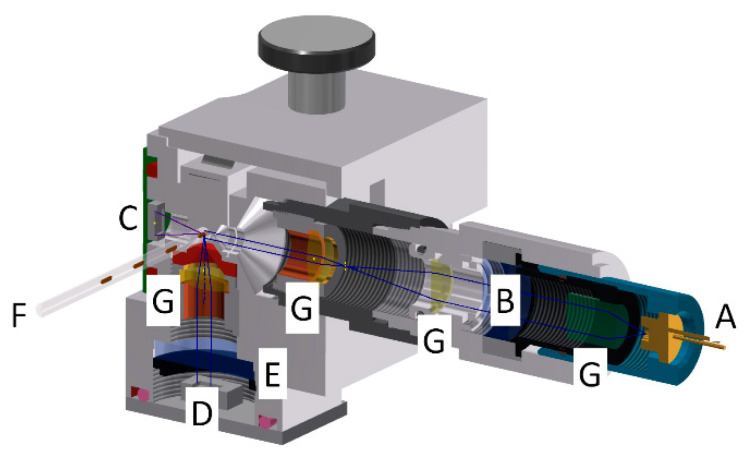
Schematic view on the photo-fluorimeter core unit. The design allows the simultaneous recording of absorbance and fluorescence signals from a passing droplet. (A) Excitation laser diode; (B) fluorescence excitation filter; (C) photodiode for absorbance measurement; (D) Photosensor (avalanche diode) for the detection of fluorescence light; (E) fluorescence emission filter; (F) FEP-tube for guiding the droplet sequences; (G) optical beam forming elements. The excitation wavelength of the laser diode (A) and filters (B + E) must be adapted to the desired fluorescence wavelength.

The desired readout for microbial dose–response assays in homogenous cultures is the broth cell density. The broth cell density is traditionally determined by turbidity measurements. From a physical point of view, the attenuation of light is called absorbance or optical density (OD) and can be measured at different wavelengths. Depending on the used medium, a calibration curve of cell density as a function of absorbance must be recorded for quantitative measurements. Alternative options of optical detection are fluorescence measurements. The fluorescence readout is an established method for many different assays using markers or fluorescent labels. The proliferation of fluorescent cells can be detected very sensitively but requires appropriately labeled organisms. However, depending on the desired assay protocol, cell-derived autofluorescence can also provide information about the microorganisms and their metabolic activity. The analysis of both parameters for a single droplet from complete droplet sequences can be challenging if one readout channel has no signal. In this case, a proper indexing of the individual droplet may fail and the correlation between data sets which are recorded by two different sensors becomes a challenge. The accurate mapping of one droplet index to the signals is nearly impossible if one sensor has intensity close or equal to the background noise. To overcome this problem, we developed a combined photo-fluorimetric sensor which acquires data from one point. For this purpose, a laser diode/photodiode arrangement was enhanced by a perpendicular oriented photo-sensor with band- or long pass filter. Thus, the absorbance can be detected by the photodiode and the fluorescence by the perpendicular oriented photosensor. The excitation wavelength can be varied by simple changing the laser diode and the filters. In Figure 7 the core of the sensor is schematically shown.

## 4. Materials and Methods

### 4.1. Microorganisms and Chemicals

For the sensor calibration prokaryotic bacteria *Bacillus sporothermodurans* PL01, *Escherichia coli* RV308, *Corynebacterium glutamicum* WT (ATCC13032) as well as the green algae *Chlorella vulgaris* were used. *Bacillus megaterium* was used for dose–response screenings against antibiotics. For the pre-cultivation of all prokaryotic bacteria, a single colony of bacteria was transferred from agar plates into an Erlenmeyer flask filled with 10 mL medium and cultivated for 24 h at 28 °C while being shaken. *Bacillus sporothermodurans* PLO1 and *Bacillus megaterium* were cultivated in actinomyceten minimal medium (AM medium), which consist of 0.5-g/L L-asparagine; 0.5-g/L K_2_HPO_4_; 0.2-g/L MgSO_4_∙7H_2_O; 0.01-g/L FeSO_4_ and 20-g/L D-glucose). *Escherichia coli* strain RV308 was provided by Hans Knöll Institute, Jena, Germany. For the experiment, the *E. coli* was cultivated in AM medium as well as in lysogeny broth (LB) complex medium (10-g/L tryptone; 5-g/L yeast extract and 10-g/L NaCl). *Corynebacterium glutamicum* WT (ATCC13032) was obtained from university of Bielefeld, group Multiscale Bioengineering. A defined medium CGXII consisted of 20-g/L (NH_4_)_2_SO_4_; 1-g/L K_2_HPO_4_; 1-g/L KH_2_PO_4_; 5-g/L urea; 13.25-mg/L CaCl_2_·2H_2_O; 0.25-g/L MgSO_4_·7H_2_O; 42-g/L MOPS; 10 mg/L FeSO_4_·7H_2_O; 10-mg/L MnSO_4_·H_2_O; 0.02-mg/L NiCl_2_·6H_2_O; 0.313-mg/L CuSO_4_·5H_2_O; 1-mg/L ZnSO_4_·7H_2_O; 0.2-mg/L biotin; 30-mg/L protocatechuic acid and 10-g/L D-glucose. The pH of the medium was adjusted to 7.0 with 1-M NaOH.

Green algae *Chlorella vulgaris* was obtained from the Technical University Karlsruhe, Institute of Applied Biosciences and Food Chemistry. The algae were pre-cultivated for 10 days at 25 °C in IGV medium under constant shaking and 16 h light to 8 h dark rhythm illumination with a light intensity of about 5000 Lux. The IGV medium for algae incubation was composed of 0.5-g/L KNO_3_; 0.34-g/L KH_2_PO_4_; 0.5-g/L MgSO_4_∙7H_2_O; 0.014-g/L FeSO_4_∙7H_2_O; 0.05-g/L EDTA; 0.74-mg/L ZnSO_4_∙7H_2_O; 57 µg/L H_3_BO_3_; 0.238-mg/L CoSO_4_∙7H_2_O; 0.236-mg/L CuSO_4_∙5H_2_O; 7.21-mg/L MnSO_4_∙H_2_O and 0.2464-mg/L Co (NO_3_)_2_∙6H_2_O. The pH of the medium was adjusted to 6.7 with 1-M NaOH.

All chemicals used for media preparation were purchased from (Sigma-Aldrich, Darmstadt, Germany). The following chemicals were also applied: tetracycline, chloramphenicol (Sigma-Aldrich, Darmstadt, Germany), perfluoromethyldecalin (PP9, F2-Chemicals, Ltd., Preston, United Kingdom), Orange G (Merck KGaA, Darmstadt, Germany) and Cochineal red A (VWR International, LLC, Radnor, PA, USA).

### 4.2. Fluidic System Characterization

The overall system performance, droplet generator accuracy and syringe pump controlling procedures must be proven before a screening series. Beside the proper workflow, the most important criterion is the achievable precision of the droplet composition. Therefore, the concentration inside the droplets was measured by the help of dyes as model for effectors.

### 4.3. 1D-Dose Response Screening

**Figure 8:**
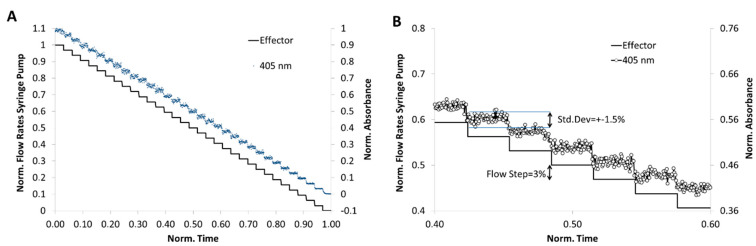
Analysis of the droplet composition accuracy regarding to the desired programmed composition. One-dimensional program with stepwise varied concentrations of a reporter dye (cochineal red A). A sequence with about 1700 segments was generated and the dye concentration determined by absorbance measurement using the photo-fluorimetric sensor module. (**A**) fluidic program (expected value, solid line) and measured value (blue dot line) for the droplet generation in 33 concentration steps; (**B**) detailed cutout of programmed (solid line) and measured values (dot line).

For a 3% concentration step resolution a flow rate program, as shown in Figure 8A, was applied. 3 mM cochineal red A was chosen for this simulation experiment and the segment-intern dye distribution was detected by microflow-through photometer with a peak wavelength at 505 nm. Through the application of the program combined with the newly developed droplet generator, a segment sequence with about 1000 droplets was generated. The correlation between the programed and measured composition showing the high reliability of the method (Figure 8). In Figure 8B the expected concentration (solid line) and the effectively obtained relative dye concentration (dot line) is shown at about 50% dilution. The result shows that the effectively achieved dye concentration deviates only slightly (±1.5%) from the desired value.

### 4.4. 2D-Dose Response Screening

**Figure 9:**
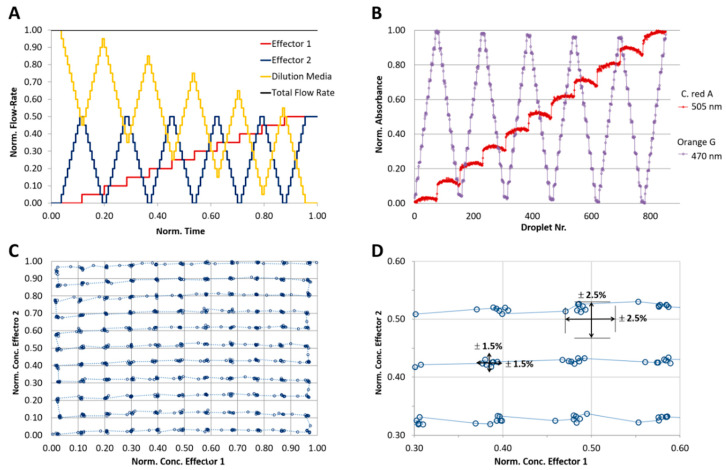
Analysis of the droplet composition accuracy regarding to the desired programmed composition. Two-dimensional program with stepwise varied compositions of two reporter dyes (C. red A and Orange G) A sequence of about 800 droplets was analyzed by two wavelength photometry. Absorbance of the two dyes (505 and 470 nm) were detected independently. (**A**) Syringe pump program for the generation of 2D concentration spaces in 11 concentration steps resulting in 121 concentration points; (**B**) measured droplet absorbance of reporter dyes; (**C**) segment composition accuracy based on the measured dye concentration combinations as normalized absorbance/absorbance plot; (**D**) detailed cutout of measured values; (C) data shows that the precision of the desired droplet composition is about ±2.5 and can be achieved with a deviation of about ±1.5%.

For the analysis of the achievable precision of a 2D concentration space, two effector model dyes must be used. A flow program with stepwise increase of the dye concentration was applied. To achieve a concentration resolution of 10% each, eleven different concentration steps (0, 10…100% of max. effector concentration) were programmed, resulting in 11^2^=121 combinations. Two anionic indicator dyes, 1-mM cochineal red A and 1-mM Orange G were used as aqueous solutions. The total flow rate of the aqueous phase (sum of two effector solutions and dilution medium) was set to 64 µL/min and the flow rate of the carrier liquid was set to 136 µL/min. After the droplet formation, they were transported with a constant flow rate (200 µL/min) through a microflow-through photometer consisting of two light emitting diodes with peak wavelengths of 470 and 505 nm for monitoring the absorbance of the indicator dyes. Through the application of the program shown in Figure 9A, a sequence of about 800 droplets was generated. The achieved normalized absorbance of both dyes is shown in Figure 9B. The effectively obtained 2D-dye distribution deviates marginally (±2.5%) from the expected value.

### 4.5. Photo-/Fluorimetric Sensor Calibration for Different Microbial Cultures

Important task for the analysis of microbial growth behavior in homogenous cultures is the calibration of the used sensor. For the cell number correlated optical density measurement the sensor module was used to determine calibration curves for different exemplary microorganisms. Therefore, dense broth cultures of different microorganisms were prepared in different media. The cultures could grow up to the stationary phase and the cell density was determined by counting the objects in a counting chamber by microscopy.

For the absorbance measurement (optical density), the minimal detectable cell number is a critical point. If we assume that relevant dose–response data are generated from cell suspensions with a cell density range of 10^5^ to 10^9^ cells/mL, the use of low volume droplets of such cell densities leads to low cell numbers per droplet. Table 1 gives an overview on the cell numbers per droplet when a culture broth is compartmentalized into different volumes. In dose–response experiments, the proliferation rate without effector is the kinetic reference. Depending on the organism under investigation, the cell density can reach numbers of up to 10^10^ cells/mL or even more.

**Figure 10:**
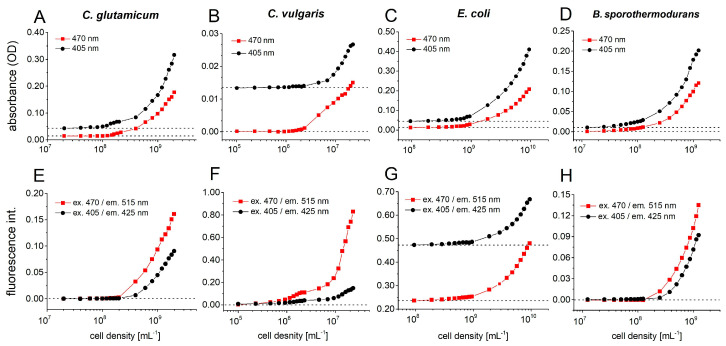
Sensor calibration for different microbial cultures cell density. The dependence of absorbance and autofluorescence intensity on inoculum cell density was recorded for different microorganisms. The signals were calculated as mean value of 30 droplets with an average size of 440 nL, using the multi-channel photo-fluorimeter device configuration: absorbance at 470 nm and 405 nm, fluorescence channels: ex. 470/em. 515 nm and ex. 405/em. 425 nm; (**A**) Absorbance of *C. glutamicum*; (**B)** absorbance of *C. vulgaris*, (**C**) absorbance of *E. coli RV308*, (**D**) absorbance of *B. sporothermodurans* PL01, (**E**) autofluorescence of *C. glutamicum*, (**F**) autofluorescence of *C. vulgaris*, (**G**) autofluorescence of *E. coli RV308*, (**H**) autofluorescence of *B. sporothermodurans* PL01.

Four different microorganisms were chosen to confirm the feasibility of cultivation in microfluid segments (Figure 10). The signals were determined from the mean value of 30 droplets with an average size of 440 nL, using a multichannel photometer (470 nm and 405 nm) and fluorimeter set (ex. 470/em. 515 nm and ex. 405/em. 425 nm) (see Figure 10).

The dependency of the absorbance and autofluorescence intensity on the cell density of *Corynebacterium glutamicum* WT (ATCC13032) is shown in Figure 10A,E. *C. glutamicum* is a soil bacterium, which was described as a short, aerobic, Gram-positive rod that are capable of producing significant amounts of different L-amino acids. *C. glutamicum* is an important industrial microorganism and is currently used for production of about 10^6^ tons of this amino acid annually, which is used as a flavoring agent [43]. From a cell density of 10^8^ mL^−1^ onwards, the growth of *C. glutamicum* can be detected using dual photo- and fluorimeter.

Alga *Chlorella vulgaris* is an ecologically important organism and is frequently used for assessing the aquatic toxicity of various chemicals [44]. Figure 10B shows a correlating trend for both photometer signals (470 and 405 nm), from a cell density of 2·10^6^ mL^−1^ onwards, the optical density increases continuously. Compared to the photometer signal, the fluorimetric signals of *chlorella* are more sensitive. Already beginning from a cell density of 5·10^5^ mL^−1^ (corresponds to ca. 220 cells each 440-nL droplet), the autofluorescence intensity at 470/515 nm channel increases. The fluorescence channel 470/515 nm had a much faster increase in intensity with rising cell numbers than the 405/425 nm channel, because the maximum excitation of fluorescent chlorophyll *b* is at 470 nm (Figure 10F).

The Gram-negative, facultative aerobic bacterium *Escherichia coli* is one of the best-studied prokaryote model organisms [45]. We have already used this model organism in many micro toxicological studies before [46,47]. In the case of *E. coli*, a high background fluorescence intensity was observed due to the LB-complex medium. Starting at a cell density of 10^8^ mL^−1^, a steady increasing signal was observed in both photo- and fluorimeter channels (Figure 10C,G).

*Bacillus sporothermodurans* PL01 is a bacterium which produces highly temperature-resistant endospores [48]. In contrast to green algae *C. vulgaris*, the photometer signal of PL01 increases from a cell density of 2·10^7^ mL^−1^ on, whereas the autofluorescence intensity can be measured beginning from a cell density of 2·10^8^ mL^−1^ (Figure 10D,H).

### 4.6. Dose–Response Screening of B. megaterium Growth Against Antibiotics

The earlier presented system and methods were applied to analyses growth kinetics and nonlinear dose response relations exemplary. *Bacillus megaterium* was chosen as example to show the growth kinetic analysis. *Bacillus megaterium* is an aerobic, Gram-positive, endospore-forming, rod-shaped soil bacterium. This species is commonly used in the laboratory as well as in industry. It can produce a variety of proteins and sources for bioremediation. The biotechnological investigation of *Bacillus megaterium* provides a wide range of different proteins that can be used for important medical, scientific and industrial advances [49]. We analyzed the growth kinetic *B. megaterium* as response to different antibiotic doses exemplarily.

The flow rates of the carrier liquid PP9 and cell suspension were set at 136 and 32 µL/min, respectively. The flow rates of the effector solution and cultivation medium were varied depending on the concentration combinations, but were adjusted to a total flow rate of 32 μL/min. Thus, the overall flow rate of the segment generation process was kept constant at 200 μL/min. An initial cell density of about 10^7^ spores/mL corresponding to about 2200 cells per segment (segment size about 440 ± 10 nL) was applied. The final concentration of the effector solution for tetracycline and chloramphenicol were set at 7 µg/mL for 1D- and 3.5 µg/mL for 2D-screening. In total, the experiments were repeated three times and for each experiment about 630 droplets for individual effector screening and about 1400 droplets for the screening of binary mixtures were generated. For the screening of binary mixtures, the program shown in Figure 9A. was applied. For each concentration step of the 121 combinations about 11 repeats were realized. This redundancy was applied to prove the reliability of measurements and to identify stochastic effects eventually caused by the small volume and the small number of initial cells for incubation. After formation and measurement, the sequence was directly transported into the tube coils. Thereafter, the tube coils were used for either static or dynamic incubation at 28 °C. Static incubation means that the coils were placed in the incubator after generation and measured after a certain point in time (e.g., 2 days). Dynamic incubation (see Figure 4) means that the sequence was continuously transported back and forth through the sensors at a flow rate of 30 µL/min after generation, so that a time-resolved dose–response relationship (about each hour) could be determined.

Linear programs providing a continuous increase in tetracycline concentrations were applied in separate experimental screening runs. Fluid segments of about 450 nL were generated and inoculated with about 2500 cells per segment. The growth was evaluated after each hour by measuring the optical density and autofluorescence by micro flow-through dual photo-fluorimetry.

Tetracycline (TC) is a broad spectrum antibiotic used for the prevention and control of a variety of infectious diseases. The first tetracycline was isolated from *Streptomyces aureofaciens* in 1947. TC acts bacteriostatically on Gram-positive and numerous Gram-negative bacteria as well as spirochetes [50]. They bind to the 30S ribosomal subunit of the 70S ribosome of the prokaryotes and prevent the attachment of the aminoacyl-tRNA complex to the ribosomal acceptor site. This leads to the inhibition of the elongation of growing peptide chains. TC also inhibits cell wall biosynthesis.

**Figure 11:**
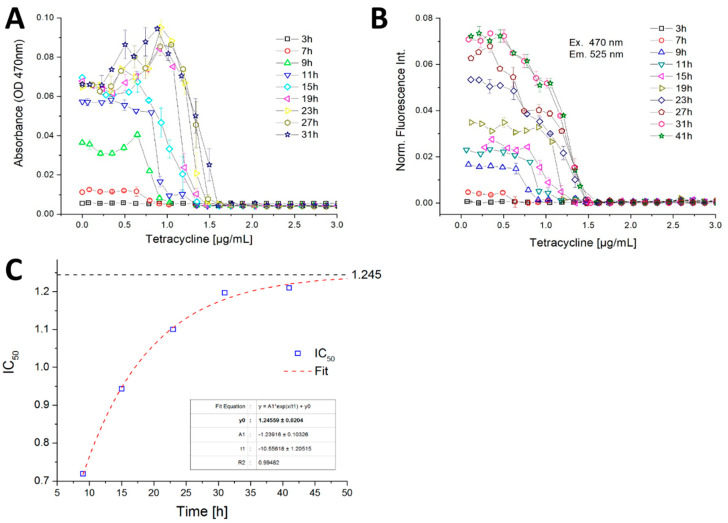
Dose–response curves with high concentration- and temporal-resolution based on the (**A**) optical density and (**B**) autofluorescence intensity of *B. megaterium* against tetracycline obtained in micro-fluid segment sequences with continuously varied effector concentrations; (**C**) IC50 value development during the incubation time from 7 to 41 h. Asymptotic fit reveals an IC50 value of about 1.245 µg/mL.

A sharp transition between growth and growth inhibition was found in the case of *B. megaterium* against TC by measuring the optical density (Figure 11A). In this experiment, after 19 h of incubation the optical density shows a non-monotonous function with considerably higher values (about 0.1 abs) in the sub-lethal concentration range of 0.6–1.2 µg/mL. At concentrations beyond 1.6 µg/mL no more growth was detected during the observation timespan. This sharp transition between growth/non-growth and the sub-lethal stimulation effect was observed only in the photometric channel (Figure 11A). The fluorimetric measurements showed a gradual decrease of fluorescence intensity with increasing TC (Figure 11B). Both photo- and fluorimetry signals showed no growth above a TC concentration of 1.6 µg/mL. The reason for this sub-lethal stimulation effect can either be attributed to morphologic changes or stress induced chromophore production by *B. megaterium*. The IC50 value, defined as the concentration which cause 50% reduction in autofluorescence intensity of *B. megaterium* compared with the controls, was determined to be about 0.7 µg/mL after 7 h of incubation. Between 7 and 31 h incubation time the IC50 value increases continually. After 31 h incubation time, the fluorescence intensity reached a stationary level, so that the IC 50 value was kept constant at approximately 1.24 µg/mL (Figure 11C).

**Figure 12:**
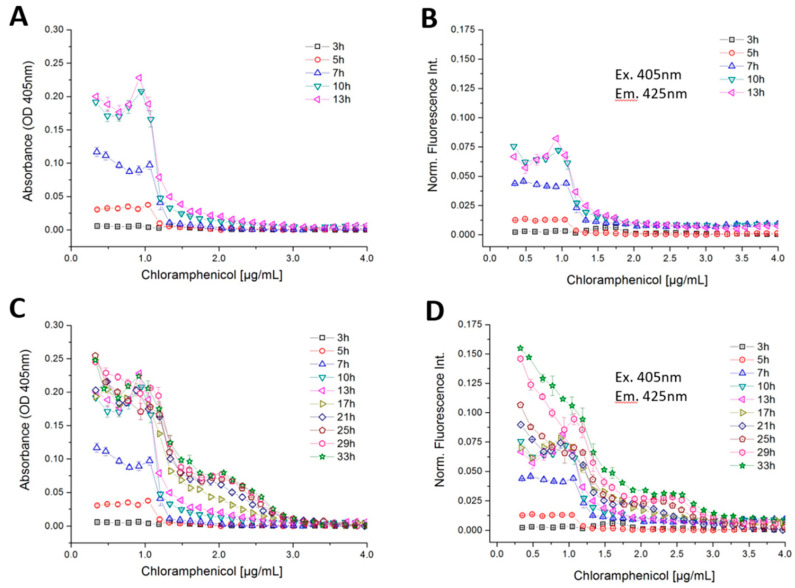
Highly concentration and time-resolved dose–response curves of *B. megaterium* against chloramphenicol based on (**A**) optical density of an incubation time of 3 to 13 h, (**B**) autofluorescence intensity of an incubation time of 3 to 13 h, (**C**) optical density of an incubation time of 3 to 33 h, (**D**) autofluorescence intensity of an incubation time of 3 to 33 h.

A higher tolerance was found with *B. megaterium* when chloramphenicol (CMP) was applied (Figure 12). Chloramphenicol is classified as a bacteriostatic antibiotic, which is produced naturally by *Streptomyces venezuelae*. It inhibits the formation of peptide bonds during the biosynthesis of proteins at the ribosomes [51]. In case of CMP, the non-linear response is depicted. Three different types of growth response with respect to different concentration ranges of CMP were found in the highly time- and concentration-resolved dose–response functions (Figure 12). Normal growth occurred at up to 1-µg/mL CMP and a complete suppression of growth was observed above 3 µg/mL for *B. megaterium*. At the beginning of the incubation time (3–13 h), both the photometric and fluorimetric signals showed strongly enhanced growth at a CMP concentration of 0 to 1 µg/mL and above 1 µg/mL both signal decreases sharply (Figure 12A). Up to 13 h incubation time, *B. megaterium* grows according to usual growth kinetics (see Figure 12D). After a certain time of incubation, however, a second growth phase at higher CMP concentrations takes place. Between the incubation time of 17–33 h, the optical density did not change significantly up to a CMP concentration of 1 µg/mL, while autofluorescence intensity increases continuously. After an incubation period of 17 h, a further growth of *B. megaterium* between 1–3-µg/mL CMP concentration was observed. This leads to a lower step (second shoulder) in the optical density and autofluorescence response curves at lower concentrations and to a steeper decline at higher concentrations in case of a cultivation time between 17 to 33 h of growth (Figure 12C,D). A sharp transition between full growth and reduced growth was found at 1.25-µg/mL CMP. The last measurement run, after 33 h of incubation, yielded an autofluorescence signal that was more than two times greater than it was when the incubation lasted just 17 h. A limited growth was only observed between 1.25 and 3-µg/mL CMP (Figure 12D).

### 4.7. 2D-Combination Effects of Two Antibiotics on the Growth of B. megaterium

To demonstrate that the described system is applicable for 2D does-response studies we used a binary effectors approach to assay combination effects exemplary. Therefore, the combined effects of the two above mentioned antibiotics (TC and CMP) on the proliferation of *B. megaterium* were assayed. The mechanisms of the action of both antibiotics are well understood, but there is a lack of information about possible interferences between both may leading to nonlinear response.

**Figure 13:**
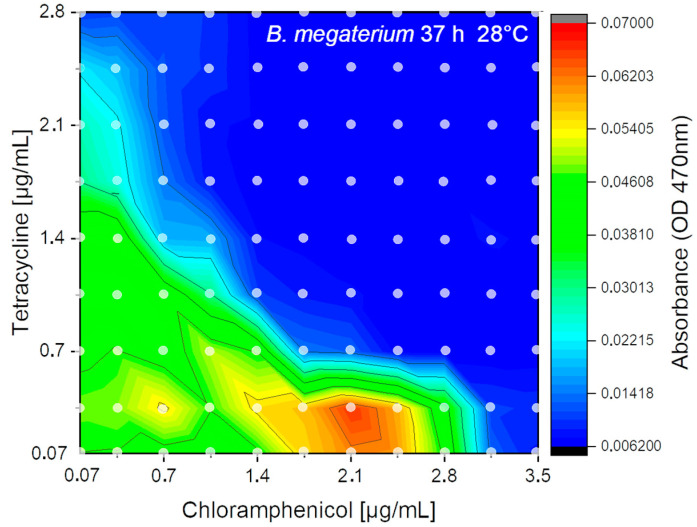
2D-isobolic plot of *B. megaterium* cell density signal (OD470) recorded after 37 h incubation with a combination of two different antibiotics (tetracycline (0.05–2.8 µg/mL), chloramphenicol (0.05–3.5 µg/mL)). Dots indicating measured data points averaged from about 12 droplets.

The flow-rate program as proven in Figure 9 was applied. For the experiment, each combination of effectors was generated with 11–12-fold redundancy. Hence, 11–12 single droplets for each of the 121 concentration points were generated resulting in about 1400 droplets. In total about 650 µL of liquids were consumed (1440 droplets each 440 ± 10 nL). The received results after fluidic processing, 37 h of incubation and sensory analysis are shown as isobolic-plot in Figure 13. In the 2D experiment the higher chloramphenicol tolerance (about 3 µg/mL) of B. megaterium compared to the tetracycline (about 1.6 µg/mL) was confirmed. Hence, the 2D-experiments showed nearly the same inhibition concentration compared to single effector screening. Furthermore, the 2D experiment showed that sublethal concentrations of chloramphenicol (1.8–2.5 µg/mL) combined with a lower concentration of tetracycline (0.05–0.4 µg/mL) lead to an enhanced sensor signal which indicates a stimulated bacterial growth by a factor of 1.5–2. This already known effect is due to higher transcription rates of the inducible genes for enzyme synthesis in response to failing transcriptional co-repressors by moderate inhibition of protein synthesis [52]. However, no growth stimulation of chloramphenicol was observed at sub lethal concentration of tetracycline. Furthermore, the isobolic plot clearly shows an additive effect of both antibiotics on *B. megaterium* growth.

## 5. Discussion and Conclusions

The response of microbial growth on different effectors is often not a simple linear relation and each has its own kinetics. In particular, if unknown organisms, new media or microbial mixtures are investigated a complex response can be expected. Obviously, the growth kinetics is a multi-parameter problem and will be governed not by the investigated effector but also by media and nutrition parameters. Even simple MIC-analysis of a single effector can show a non-linear response. We proved the presented screening system to analyze the growth kinetic of *Bacillus megaterium* under antibiotic (tetracycline) stress. The repetitive measurement of the droplet sequence revels that even slow bacterial growth was suppressed only at concentrations above 1.25 µg/mL. However, the steady IC50 value was reached only after 50 h in this case (Figure 11C). The comparison between photometric and fluorimetric sensor signals revels a sub inhibitory stimulation of the microorganism by the antibiotic (Figure 11A). This effect was only observed in the photometric signal but not in the fluorimetric channel. This allows the interpretation that the Bacillus changes his morphology under stress but produces no fluorescent metabolites which increase the fluorimetric sensor signal. This example shows the requirement for kinetic analysis to receive valuable IC50 values. Even sub-inhibitory effects can be only studied if time and concentration resolved data of different optical channels are available. As an example, for a non-linear dose response behavior the inhibitory effect of chloramphenicol CMP on the growth of *Bacillus megaterium* was analyzed. Here two clear distinguishing response cases can be observed. A usual growth kinetic was observed within the first 13 h revealing an inhibitory effect in concentration range beyond 1.25 µg/mL (Figure 12A,B). A weak sub-lethal stimulation can be found at about 1.0 µg/mL concentration which was observed in both channels. But after 17 h, a second growth phase arise. The *Bacillus megaterium* was able to proliferate slowly even in a concentration range between 1.25 up to 3.0 µg/mL (Figure 12C,D). The second growth phase was observed in both sensor channels. However, to suppress the bacterial growth efficiently a dose beyond 3.0 µg/mL tetracycline is necessary. Such non-linear secondary growth effects can be observed only if the growth kinetic is analyzed carefully for a prolonged time. A logic following up question is which effect will have the combination of both antibiotics. The droplet-based assay results of the combined effects are shown in Figure 13. The isobolic plot reveals the same IC50 values for the single antibiotic in presence of only low concentrations of the second. A clear synergistic effect was found as well as region of stimulated growth. Concluding shows the presented examples the capability and advances of the presented modular droplet-based system for the analysis of nonlinear dose–response screening runs.

## Figures and Tables

**Table 1:** Cell numbers per droplet in dependence on appropriate inoculum densities.

Inoculum	Compartmentalized in Droplets of Volume:						
Cell/mL	1 nL	10 nL	100 nL	500 nL	1 µL	10 µL	100 µL
1							
10							1
100						1	10
1000				1	1	10	100
10,000			1	5	10	100	1000
100,000		1	10	50	100	1000	10,000
1.0 × 10^6^	1	10	100	500	1000	10,000	100,000
1.0 × 10^7^	10	100	1000	5000	10,000	100,000	1.0 × 10^6^
5.0 × 10^7^	50	500	5000	25,000	50,000	500,000	5.0 × 10^6^
1.0 × 10^8^	100	1000	10,000	50,000	100,000	1.0 × 10^6^	1.0 × 10^7^
1.0 × 10^9^	1000	10,000	100,000	500,000	1.0 × 10^6^	1.0 × 10^7^	1.0 × 10^8^
5.0 × 10^9^	5000	50,000	500,000	2.5 × 10^6^	5.0 × 10^6^	5.0 × 10^7^	5.0 × 10^8^
